# Outcome assessment of the VADO approach in psychiatric rehabilitation: a partially randomised multicentric trial

**DOI:** 10.1186/1745-0179-2-5

**Published:** 2006-04-03

**Authors:** Rosaria Pioli, Michela Vittorielli, Antonella Gigantesco, Giuseppe Rossi, Luigi Basso, Chiara Caprioli, Chiara Buizza, Angela Corradi, Fiorino Mirabella, Pierluigi Morosini, Ian RH Falloon

**Affiliations:** 1 Psychiatry Rehabilitation Unit, IRCCS "Centro S. Giovanni di Dio-Fatebenefratelli", Brescia, Italy; 2 Mental Health Unit, National Centre of Epidemiology, Surveillance and health Promotion, Italian National Institute of Health, Rome, Italy; 3 Psychiatry Rehabilitation Unit, Grieserhof clinic, Bolzano, Italy; 4 University of Auckland in Auckland, New Zealand

## Abstract

**Background:**

Recent studies on representative samples of psychiatric services have shown that low proportions of cases received effective rehabilitation interventions. The following are likely to be the most important causes: the scarcity of mental health workers trained in social and work skills strategies and the absence of a structured framework to formulate rehabilitation practices.

The aim of this study was to assess if a specific structured planning and evaluation manual, called VADO (*Valutazione delle Abilità e Definizione degli Obiettivi *– in english: *Skills Assessment and Definition of Goals)*, is more effective than routine interventions in reducing disability in patients with schizophrenia.

**Method:**

Each of 10 mental health services were invited to recruit 10 patients with a schizophrenic disorder. Altogether 98 patients were recruited. Of these, 62 patients were randomly allocated to the intervention/experimental or a control group. The remaining group of 36 patients was not randomised and it was considered as a parallel effectiveness study. Assessment measures at the beginning of the study and at the one-year follow-up included the FPS scale of social functioning and the BPRS 4.0. Between group (VADO vs. Routine) and time effects were examined with ANOVA, Chi-square or Fisher exact. Clinical "improvement" was defined as an increase of at least ten points on the FPS or a decrease of at least 20% on BPRS scores.

**Results:**

31 of the 62 randomized patients received the experimental interventions, while 31 followed the routine ones. At follow-up, the experimental group showed statistically and clinically greater improvements in psychopathology and social functioning.

Better outcomes of both social functioning and symptom severity were observed in non randomised patients (parallel effectiveness study).

**Conclusion:**

The results suggest that setting personalised and measurable objectives, as recommended by the manual, can improve the outcome of rehabilitation of severe mental disorders. Better outcomes in the parallel effectiveness study could be attributed to the greater confidence and enthusiasm of staff in centres where the VADO approach originated.

## Background

The results of a recent study on a representative sample of Italian psychiatric services [1] have shown that only 35% of patients with schizophrenia who live with their own family follow a rehabilitation programme and that such rehabilitation programmes include the setting of personalised objectives in only 66% of cases. Therefore, only 23% of patients may receive effective rehabilitation interventions. Similar low proportions of cases receiving evidence-based psychosocial interventions have been observed in the US [2]. Among the causes, the following are likely to be the most important: psychodynamic orientation of many rehabilitation managers, the scarcity of mental health workers trained in social and work skills strategies and the absence of a structured framework to formulate rehabilitation practices [3].

In 1998 the rehabilitation centre of the Fatebenefratelli Institute of Brescia, in collaboration with the Italian National Institute of Health and the Institute of Psychiatry of the University of Napoli, published a short manual for the planning and evaluation of rehabilitative interventions in psychiatric facilities [4]. The principles were derived from the Boston University approach [5], introduced in Italy by Marianne Farkas. The handbook was called VADO (*Valutazione delle Abilità e Definizione degli Obiettivi *– in english: *Skills Assessment and Definition of Goals)*.

The effectiveness of the VADO approach was assessed in a pilot study of 45 patients with schizophrenia, all treated in Brescia. Encouraging results in this study over a 6-month follow-up [6] raised the question of its efficacy and a multicentric controlled study was then planned.

### Aims of the study

The aim of this report is to give a full description of the controlled study and the one-year follow-up results. The main outcome criterion was social functioning; however, since the rehabilitation strategy also focussed on self-management of mental disorders, psychopathological symptoms were also considered.

## Methods

### Procedure

The study was conducted in 10 day or residential rehabilitation centres; two in Brescia, Rome and Mantova, and one in Bolzano, Bologna, Brugherio, Soresina.

In the 6 months following a VADO training course (see later), each centre recruited at least 10 patients fulfilling the following criteria: a) aged between 18 and 65 years; b) diagnosis of schizophrenic, schizoaffective or delusional disorder according to ICD-10; c) global functional score < 70 on the FPS (see later; this score corresponds to manifest difficulties in at least one key functioning area); d) absence of disabling physical diseases, psycho-organic syndromes or of mental retardation of medium to severe degree, according to the ICD-10 criteria; e) stable medication regimen.

The patients were allocated to the intervention/experimental or the control group according to a randomised sequence. The randomisation protocol was held in the Brescia central unit and the centres had no possibility to influence the allocation. Altogether 98 patients were recruited, 57 in the intervention group and 41 in the control group. However, only 62 patients were randomised (31 to the intervention group and 31 to the control group). The remaining 36 patients were not randomised because: a) 18 patients recruited by the two residential facilities of Brescia and 8 from the rehabilitation day centre of Monza were all allocated to the intervention group, in order to avoid a contamination bias, because the rehabilitation workers there were already all implementing the VADO approach; b) all 10 patients of the Bologna centre that had been originally allocated to the VADO group had to be included in the control group, because all the VADO trained workers were suddenly transferred before beginning the VADO-oriented rehabilitation process. This further subgroup of 36 non-randomised patients was considered as a parallel effectiveness study.

Patients signed an informed consent form after the study had been explained to them.

### Assessments

At the beginning of the study (T0) the socio-demographic characteristics of the patients, illness duration and hospital admissions during the last year were recorded. At T0, after 6 months (T1) and after 1 year (T2), psychopathology was measured with the Italian standardized BPRS 4.0 [7] and social functioning with the Personal and Social Functioning Scale, FPS (see later).

BPRS and FPS assessments were carried out by the VADO-trained rehabilitation workers. The assessments were checked by one independent research assistant at each centre, who interviewed the rehabilitation workers after each assessment. The research assistants were instructed not to ask anything that could reveal the treatment received by the patients. In the few cases of disagreement between the research assistant and the rehabilitation workers, the rating was made by another independent research assistant. The VADO Personal and Social Functioning Scale (FPS) is a modified version of the Social and Occupational Functioning Assessment Scale (SOFAS) [8]. As with the SOFAS, the FPS scores range from 100 (excellent functioning) to 1 (extremely severe impairment with risk for survival). The instructions for scoring the 10 points within each level are more detailed. The rater is instructed to take into account four main areas: *work and/or socially useful activities*; *family, personal and social relationships; self-care*; *aggressive and destructive behaviours*. Suicide risk is considered in the score only as much as suicidal ruminations may interfere with social-functioning. The FPS requires a brief and simple training, that is described in the VADO manual. The FPS can be easily scored by rehabilitation workers, including those with a limited psychiatric experience. It is, therefore, an instrument that may be useful to assess severity and outcome in routine practice [9]. Thirty patients were rated independently by two professionals: the non-weighted 10 levels k = 0.75.

BPRS was analysed according to the following factors: *positive (items 9–12, 14–15, and 24) and negative (items 13, 16–18, and 20) symptoms, anxiety and depression (item 1–5, and 19), mania and hostility (items 6–8. and 21–23)*.

### Approach groups

#### a) VADO approach

The aim of the VADO approach is to help the rehabilitative team to define individual rehabilitative programs, to focus on negotiating realistic specific (measurable) goals, and to routinely evaluate the attainment of those specific goals, first, within the rehabilitation unit, and subsequently, in real life. The VADO approach comprises five components: 1) assessment of 28 area of social functioning, including an area of skills, and choice of priority areas aspects compatible with so called 'global objective' (how the patient would like to live); 2) global evaluation, 3) negotiation of realistic, attainable, specific measurable objectives, 4) subdivision of specific objectives in skills and tasks; 5) maintenance and generalisation. The VADO handbook [4] provides detailed instructions on how to assess patients' disabilities and residual strengths and how to negotiate the rehabilitation program with him/her, as well as worksheets and forms. These include the Functioning Assessment (FA) to collect information covering 28 domains of patient's functioning. On the basis of the FA, rehabilitation workers score the 28 domains on a 6-point scale on the Rehabilitation Areas Form (RAF). The RAF assesses the need for rehabilitation in each domain, and whether a rehabilitation intervention is planned to meet that need.

The VADO manual does not detail rehabilitation techniques, but explicitly recommends modelling, non-verbal and verbal prompting, role-playing [10], and structured problem solving. In the self-management of mental disorders it outlines the stress-vulnerability model, identification of early signs of relapses and functional coping strategies enhancement, according to the models of Falloon [11], Fowler et al. [12], and Chadwick et al. [13]. Motivational interviewing strategies are recommended to correct misinformation about medication and to improve adherence.

#### b) Routine approach

The control treatment consisted of the usual rehabilitation activities of each participating centre. The ongoing activities were described according to the classification of "*Glossary of interventions and activities of Mental Health Departments" *[14]. Two thirds of participating centres stated that they had already a policy of personalised programs in interpersonal and social skills and all stated that they were organising some kind of social activity (group discussions, newspapers reading with comments, movie watching, and so on); two thirds of centres were also organising parties, excursions and supported holidays. The rehabilitation workers of the control patients were not trained in the VADO approach.

### Psychopharmacological treatment

No change in psychopharmacological treatment was planned for patients in either group. The VADO approach recommended providing detailed information to patients about the purpose, nature and side effects of pharmacological treatments in order to improve not only adherence, but also active participation in medication management.

### Training in the VADO approach

Before beginning the study, at least two staff members from each unit (one physician and one or more rehabilitation workers) attended a 32-hour training course (8 hours a day for 4 days). The course was based on the small groups methodology, with extensive role playing and case discussion. Participants were shown video-recorded cases and were asked to give the FPS score (see later) and fill the RAF forms independently and then to discuss any inconsistencies in their ratings. Participants did not receive no further training in rehabilitation techniques. However, because of its features, the VADO training induced to apply or consolidated the application of social skills training techniques in the program. For example, the trainer showed the group segments of videocassettes that provided examples on how to evaluate the progress toward the achievement of a specific measurable patient goal after using techniques based on social learning techniques such as demonstration, role playing, positive feedback, shaping and generalisation.

After the course, participants in the study attended one day per month supervision sessions for six months. During these sessions they discussed problems and doubts about the clinical management of cases.

### Statistical analysis

Data were analysed with SPSS 12.0. Between group (VADO vs. Routine) and time effects (T0 vs. T1; T0 vs. T2) were examined with ANOVA, Chi-square or Fisher exact tests depending on the nature of the data. Clinical "improvement" was defined as an increase of at least ten points on the FPS or a decrease of at least 20% on BPRS scores.

To identify variables associated with improvement at T2, two different multiple logistic regression analyses were performed. In the first, the FPS score was the dependent variable and we entered treatment group, age, sex, length of disease, and FPS T0 scores as independent variables. In the second, the BPRS total score was the dependent variable and treatment group, age, sex, length of disease, and BPRS total score at T0 were entered as independent variables.

## Results

### 1. Patient characteristics (see Table 1)

**Table 1:** T0. Characteristics, living conditions, previous admissions of patients initially recruited in the study. Between brackets, standard deviations.

Variables	Experimental group N = 57	Control group N = 41	Total N = 98
**Male sex (%)**	60	70	64
**Age average**	39 (10)	42 (10)	40 (10)
**Years of completed education cycles (%)**			
5 years or less	13	35	22
8 years	60	44	53
12–13 years or more	27	21	25
**Occupied (%)**	29	33	31
**Usual living conditions**			
Alone	6	15	10
With relatives	59	54	57
in residential centres	35	31	33
Duration of disorder in years *	17 (9)	21 (10)	19 (9)
**Voluntary admissions in last year**			
0	55	70	61
1	20	10	21
2 or more	15	20	18
**Compulsory admissions in last year**			
0	86	93	89
1	12	5	9
2 or more	2	2	2

statistically significant differences (P < 0.05) between the two groups.

Patients were mostly unemployed single males with a long duration of illness; two-thirds had limited education. The two groups had similar socio-demographic and clinical variables. However, duration of illness was significantly greater in the control group, which also showed a trend towards higher BPRS and lower FPS scores. The RAF domains in which problems were present in more than 75% of the patients were social activities, family life, friends and supporting relationships, self management of mental health, work and socially useful activities. At T0, the presence of problems was ascertained in 69% of the domains in the experimental group and in 73% in the control group.

### 2. Setting and achievement of rehabilitation objectives

The percentage of problem areas in which rehabilitative programs were implemented was 20% in the experimental and 13% in the control group.

In the 57 experimental group patients, 174 objectives were planned of which 122 were achieved. All experimental group patients agreed on at least one objective. 24% of objectives involved household chores; 31% participation in residential or day centre life; and 25% aimed at self management of mental health. The areas in which a significantly greater between-group difference in the decreased proportion of patients with problems were: participation in residential or day centre life, self care, self management of mental health, work and socially useful activities, and coping with emergencies.

### 3. Randomised controlled study (N = 61)

#### 3a. Patient discharges and drop-outs

During the first 6 months no patients in the experimental group and only 3 from the control group dropped out after having psychotic exacerbations. All were single women with a duration of the illness of more than 10 years. Therefore, at 6 months, T1, the study included 59 patients, 31 in the intervention group and 28 in the control group.

From 6–12 months, 2 patients were discharged from the experimental group, and did not attend the T2 assessment. In the control group, one patient was discharged and one moved to another city; they did not attend T2 assessment. Therefore, the one-year assessment, T2, included 55 of 62 original patients (89%), 29 in the intervention group and 26 in the control group.

#### 3b. Change in social functioning (see Table 2 and Figure 1)

**Table 2:** Percentages of improved patients at 12-month (T2) follow-up versus T0 in the randomised experimental and control groups (N = 55)

Variables			
	**Experimental (N = 29)**	**Control (N = 26)**	***P value ***

**BPRS positive symptoms**	34.5	3.8	0.00
**BPRS negative symptoms**	37.9	19.2	0.13
**BPRS mania/hostility**	31.0	7.7	0.04
**BPRS anxiety/depression**	34.5	23.1	0.35
**BPRS overall**	31.0	7.7	0.04
**FPS**	51.7	42.3	0.48

**Figure 1:**
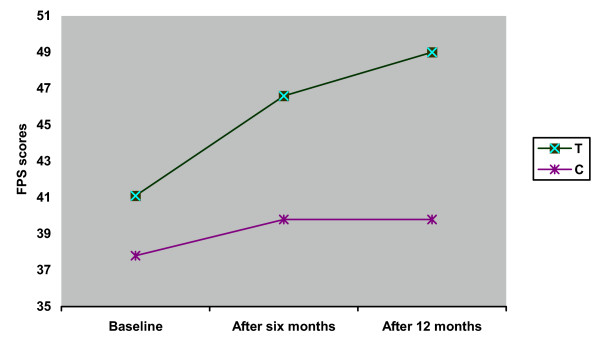
Average FPS scores at baseline, after 6 months, and after 12 months in the treated (T) and control (C) groups.

From the 31 experimental group patients that completed T1 assessments, 7 (22.6%) improved clinically compared to 3 (10.7%) of the 28 control patients (chi square = 1.472, p = 0.22). At T2, 15 of the 29 experimental group (51.7%) and 11 of 26 (42.3%) controls were improved (chi square = 0.488) (Table 2).

At T0, mean FPS scores in the experimental group were 41.1 ± 12.8 and in control group were 37.8 ± 13.4 (t = -0.984, df = 57, p = 0.33). At T1, the FPS increased in both (46.6 ± 13.1 versus 39.8 ± 14.5; t = -1.887, df = 57, p = 0.06). At T2, a the experimental group showed further improvement compared to no further improvement in the control group (49.0 ± 14.3 versus 39.9 ± 14.3; t = -2.360, df = 53, p < 0.05) (Figure 1).

#### 3c. Changes in psychopathology (see Table 2 and Figure 2)

**Figure 2:**
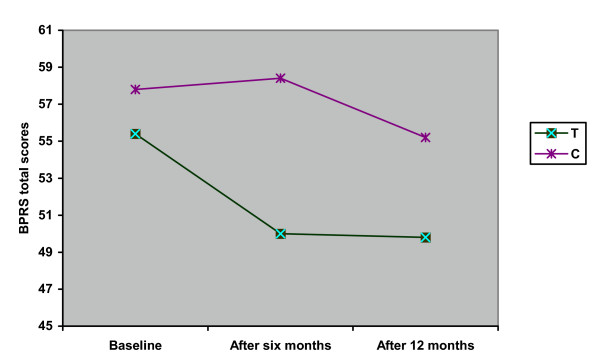
Average total BPRS scores at baseline, after 6 months, and after 12 months in the treated (T) and control (C) groups.

### BPRS global score

Significant differences between groups were found at T1 (chi square = 3.641, p = 0.06) and T2 (Table 2).

At T0, BPRS total scores in the experimental and control groups were respectively 57.8 ± 21.2 and 55.4 ± 20.3 (t = 0.445, df = 57, p = 0.66). At T1, the BPRS decreased in the experimental group (50.3 ± 21.1) and increased in the controls (58.4 ± 21.1) (t = 1.439, df = 57, p = 0.16). At T2, both groups had improved (49.8 ± 20.7 versus 55.2 ± 21.0; t = 0.965, df = 53, p = 0.34) (Figure 2).

In the multiple logistic regression analysis, a test of the full model with all five independent variables was significant (chi square = 11.5, df = 5, p < 0.05). According to the Wald criterion, the only strong association with improvement was being an experimental group patient (odds ratio: 9.67, 95% CI: 1.39 -67.08).

### BPRS positive symptoms

Significant differences between groups were found at T1 (chi square = 4.647, p < 0.05), and T2 (chi square = 8.042, p <0.01).

### BPRS negative symptoms

At T1, difference between the groups approached significance (chi square = 3.487, p = 0.06). But at T2, no significant difference was observed.

### BPRS mania/hostility

Significant difference between groups was found at T2 (chi square = 4.668, p < 0.05), but not at T1.

### BPRS anxiety/depression

No statistically significant differences were found between the groups at follow-up.

### 4. Not randomised controlled study (N = 36)

#### 4a. Patient discharges and drop-outs

During the first 6 months no drop-outs occurred in either group. At 6 months, T1, the study included 36 patients, 26 in the intervention group and 10 in the control group.

From 6–12 months, 2 patients were discharged from the experimental group, and did not attend the T2 assessment; another dropped-out after a psychotic exacerbation. In the control group, one patient had a psychotic exacerbation. Therefore, the one-year assessment, T2, included 32 of the 36 original patients (89%), 23 in the intervention and 9 in the control group.

#### 4b.Change in social functioning (see Table 3)

**Table 3:** Percentages of improved patients at 12-month (T2) follow-up versus T0 in the non randomised experimental and control groups (N = 32)

Variables			
	**Experimental (N = 23)**	**Control (N = 9)**	***P value ***

**BPRS positive symptoms**	43.5	0.0	0.03
**BPRS negative symptoms**	60.9	11.1	0.02
**BPRS mania/hostility**	34.8	11.1	0.38
**BPRS anxiety/depression**	34.8	0.0	0.07
**BPRS overall**	52.2	0.0	0.01
**FPS**	73.9	33.3	0.05

Of the 26 experimental group patients completing T1 assessments, 16 (61.5%) improved clinically compared to 0 of the 10 control patients (chi square = 11.077, p < 0.01). At T2, 17 of the 23 experimental group (73.9%) and 3 of 9 (33.3%) controls were improved (chi square = 4.545, p = 0.05).

#### 4c. Changes in psychopathology (see Table 3)

##### BPRS global score

Significant differences between groups were found at T1 (chi square = 11.077, p < 0.01) and T2 (chi square = 7.513, p < 0.05).

#### BPRS positive symptoms

Significant differences between groups were found at T1 (chi square = 9.890, p < 0.01), and T2 (chi square = 5.692, p <0.05).

#### BPRS negative symptoms

Significant differences between groups were found at T1 (chi square = 5.713, p < 0.05), and T2 (chi square = 6.432, p <0.05).

#### BPRS mania/hostility

Significant difference between groups was found at T1 (chi square = 9.890, p < 0.01), but not at T2.

#### BPRS anxiety/depression

Significant difference between groups was found at T1 (chi square = 6.092, p < 0.05), and at T2, difference approached significance (chi square = 4.174, p = 0.07).

## Discussion

This study suggests that multi-centred controlled studies of complex psychosocial interventions in routine rehabilitation settings is challenging, but feasible. The present study suggests that a structured approach to the assessment of rehabilitation needs, with specific goal setting and accountability to motivate workers to follow through with rehabilitation plans may encourage the application of evidence-based treatment approaches, and lead to improved social and clinical outcomes.

This study has several strengths:

1) It evaluated the efficacy of a structured rehabilitation intervention in clinical practice of common centres, with a low consumption of professional and financial resources and after a very brief, even if intensive, training. Most studies of the efficacy of specific rehabilitation interventions have been performed by highly specialised personnel and on highly selected patients [5,15-22].

2) Outcomes were investigated also for the patients that were discharged. All patients discharged in the first six months from the experimental group and most of those discharged in the second six months came back to follow-up assessments.

3) This was the first controlled partially randomised psychiatric rehabilitation study to be carried out in Italy, and it has stimulated further similar studies.

4) The VADO approach, which is based on the negotiation of measurable objectives that are relevant to patient's quality of life, seems to be very promising from a common sense point of view. This study seems to suggest that it is effective, at least when compared with less structured and more limited rehabilitation strategies. It should be noted that control group patients also improved their social functioning. This indicates that the control patients were not neglected, even if they usually received less attention from staff.

5) The lack of difference between randomised and non-randomised control groups shows that contamination cannot be ruled out. However, there is some evidence against it. Better outcomes of both social functioning and symptom severity in non-randomised patients could be attributed to the greater confidence and enthusiasm of staff in centres where the VADO approach originated.

6) The VADO approach is simple, requires only brief training and does not cost extra to apply. Thus, it is likely to prove cost-effective.

On the other hand, this study had a number of serious weaknesses. The optimal size of the study was not estimated a priori. The outcome measures were applied by the same rehabilitation workers who were assessing needs and setting goals and were neither independent or blind to treatment allocation. An effort was made to check the validity of each assessment by independent research assistants, but bias was highly likely. Future studies are planned with improved financial support to enable blind, independent assessment, to avoid contamination when different strategies are implemented in the same setting, by the same workers, and to improve the standardization of the psychosocial and pharmacological strategies implemented.

In the experimental group, the decrease in disability was associated with a marked improvement in psychopathological symptoms and a low rate of psychotic exacerbation. This result was somewhat unexpected because these clinical domains were not the main targets for rehabilitation. In the absence of any substantial changes in pharmacotherapy this finding may be partially explained with the inclusion of a symptom self-management module or it could be also a reflection of more attention paid by workers that used the VADO approach.

Patients treated with the VADO approach were also more likely to be discharged home or to sheltered apartments. In the one year of the study 40% were discharged in this manner and showed further improvement in their new environments. The VADO approach helps the patients decide where and how to live and attempts to provide them with the skills and confidence needed to achieve their independence as advocated by Marshall [23] and Mueser [24]. In contrast, a nationwide Italian study on the state of the Italian psychiatric residential facilities [25] showed that, although 32% of the patients were younger than 40 years of age, discharge rates were extremely low.

Finally, it may be noted that despite the comprehensive needs assessment and goal setting only a small proportion of problem issues were addressed during the one-year study period, and less than half the patients were discharged successfully. This may be seen as a limitation of the VADO approach, but perhaps may be also a reflection of the need for long-term rehabilitation programmes that continue to address the priority needs of patients for many years, albeit prioritising those needs that most impede the progress of patients towards their desired life styles. Research studies of rehabilitation programmes may need to extend for much longer periods to examine the full benefits of such approaches [26].

## Competing interests

The author(s) declare that they have no competing interests.
